# Autophagy controls centrosome number by degrading Cep63

**DOI:** 10.1038/ncomms13508

**Published:** 2016-11-21

**Authors:** Yuichiro Watanabe, Shinya Honda, Akimitsu Konishi, Satoko Arakawa, Michiko Murohashi, Hirofumi Yamaguchi, Satoru Torii, Minoru Tanabe, Shinji Tanaka, Eiji Warabi, Shigeomi Shimizu

**Affiliations:** 1 Department of Pathological Cell Biology, Medical Research Institute, Tokyo Medical and Dental University (TMDU), 1-5-45 Yushima, Bunkyo-ku, Tokyo 113-8510, Japan; 2 Department of Hepato-Biliary-Pancreatic Surgery, Tokyo Medical and Dental University, 1-5-45 Yushima, Bunkyo-ku, Tokyo 113-8510, Japan; 3 Department of Molecular Oncology, Graduate School of Medicine, Tokyo Medical and Dental University, 1-5-45 Yushima, Bunkyo-ku, Tokyo 113-8510, Japan; 4 Division of Biomedical Sciences, Faculty of Medicine, University of Tsukuba, 1-1-1 Tennoudai, Tsukuba, Ibaraki 305-8575, Japan

## Abstract

Centrosome number is associated with the chromosome segregation and genomic stability. The ubiquitin–proteasome system is considered to be the main regulator of centrosome number. However, here we show that autophagy also regulates the number of centrosomes. Autophagy-deficient cells carry extra centrosomes. The autophagic regulation of centrosome number is dependent on a centrosomal protein of 63 (Cep63) given that cells lacking autophagy contain multiple Cep63 dots that are engulfed and digested by autophagy in wild-type cells, and that the upregulation of Cep63 increases centrosome number. Cep63 is recruited to autophagosomes via interaction with p62, a molecule crucial for selective autophagy. *In vivo*, hematopoietic cells from autophagy-deficient and *p62*^*−/−*^ mice also contained multiple centrosomes. These results indicate that autophagy controls centrosome number by degrading Cep63.

## Abstract (web summary)

The ubiquitin-proteasome system is thought to be the primary regulator of centrosome number. Here, Watanabe *et al*. show that selective autophagy also plays a role in regulating centrosome number via p62-dependent recruitment of centrosomal protein 63 to autophagosomes.

## Graphical abstract

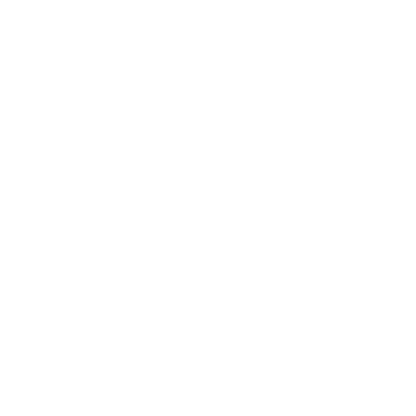

The centrosome is an organelle that plays a major role in microtubule network organization during mitosis. During prophase, centrosomes migrate to opposite poles of the cell to form the microtubule spindle apparatus on which chromosomes segregate. Centrosome number abnormalities are associated with chromosome mis-segregation and genomic instability to some extent^1^,^2^,^3^. Usually, G1 cells have one mature centrosome containing a pair of centrioles embedded in a protein-dense amorphous pericentriolar matrix. Centriole replication occurs during the S phase, and each centriole generates one daughter centriole at the G2–M phase^4^. Many protein components of the centriole, such as centrosomal protein 63 (Cep63), centrosomal protein 152 (Cep152), polo-like kinase 4 (Plk4) and spindle assembly abnormal protein 6 homologue (SAS6), have been identified as factors involved in centriole duplication^5^. Among them, Cep63 and Cep152 initially form a ring-like structure at the proximal end of the mother centriole and recruit Plk4 (refs 6, 7, 8). SAS6 and SCL/TAL1 interrupting locus (STIL) are then stabilized to form a cartwheel structure that generates the daughter centriole^9^. The number of centrioles is tightly regulated by the amounts of these centrosomal proteins mainly through the ubiquitin (Ub)–proteasome protein degradation system^10^,^11^.

Macroautophagy (hereafter referred to as autophagy) is a catabolic process in which cellular contents, including proteins, lipids and even entire organelles, are digested within lysosomes. Autophagy occurs constitutively at low levels, but is accelerated by a variety of cellular stressors, such as nutrient starvation, accumulation of abnormal proteins and organelle damage^12^. Autophagy was originally considered to be a bulk and non-selective catabolic process. However, increasing lines of evidence indicate the existence of a cargo-specific type of autophagy (termed selective autophagy) that degrades specific targets^13^. Selective autophagy operates to eliminate specific targets, such as proteins and organelles, by their delivery to autolysosomes, and functions to regulate various cellular events^14^.

The molecular machinery of autophagy has been well studied using autophagy-defective mutant yeasts and mice^15^. Activation of the unc-51-like kinase 1 (Ulk1) complex is crucial for the initiation of autophagy. Then, vesicle nucleation occurs via activation of the class III phosphatidylinositol 3-kinase (PtdIns3K) complex, which comprises PtdIns3K, Beclin 1, Vps15 and Atg14L (ref. 16). The subsequent elongation and closure of isolation membranes are mediated by two Ub-like conjugation pathways, namely, the Atg5–Atg12 pathway and the microtubule-associated protein 1 light chain 3 (LC3) pathway^15^. Atg7 is required for the conjugation of Atg12 to Atg5 as an E1-like enzyme. Conjugation of phosphatidylethanolamine to LC3 is mediated by the actions of Atg3 and the Atg5–12 complex, as E2- and E3-like enzymes, respectively. This event is coupled with the translocation of LC3 from the cytosol to the isolation membrane, and hence this translocation makes this complex a reliable marker of autophagy^15^. In the final step, ultraviolet radiation resistance-associated gene (UVRAG) and the PtdIns3K complex, excluding Atg14L facilitate autophagosome–lysosome fusion^16^. Various lines of evidence indicate that among these molecules, members of the Atg5–Atg12 conjugation system are essential for autophagy. In selective autophagy, p62 acts as cargo receptors for the autophagic degradation of substrates.

Recently, we discovered the existence of an Atg5/Atg7-independent type of autophagy (named alternative autophagy^17^), we hence extensively analysed the morphology of *Atg5*-deficient mouse embryonic fibroblasts (MEFs) by electron microscopy. During these analyses, we incidentally found that these MEFs contain multiple centrosomes. The presence of multiple centrosomes in *Atg5*^*−/−*^ MEFs led us to hypothesize that centrosome number is regulated not only by the Ub–proteasome system, but also by autophagy. Thus, we investigated whether centrosome number is regulated by autophagy, which molecules are involved in this process. As a result, we found that autophagy plays a crucial role for maintaining proper centrosome number. We also found that cells lacking autophagy contain multiple Cep63 dots and that should be the cause of the increase in centrosome number. In wild-type MEFs, multiple Cep63 dots are engulfed and digested by autophagy in a p62-dependent manner, and hence *p62*^*−/−*^ MEFs also carry extra centrosomes. *In vivo*, hematopoietic cells from autophagy-deficient and *p62*^*−/−*^ mice also contained multiple centrosomes. The present study demonstrated that autophagy controls centrosome number by degrading Cep63.

## Results

### Involvement of autophagy in centrosome number regulation

**Figure 1:**
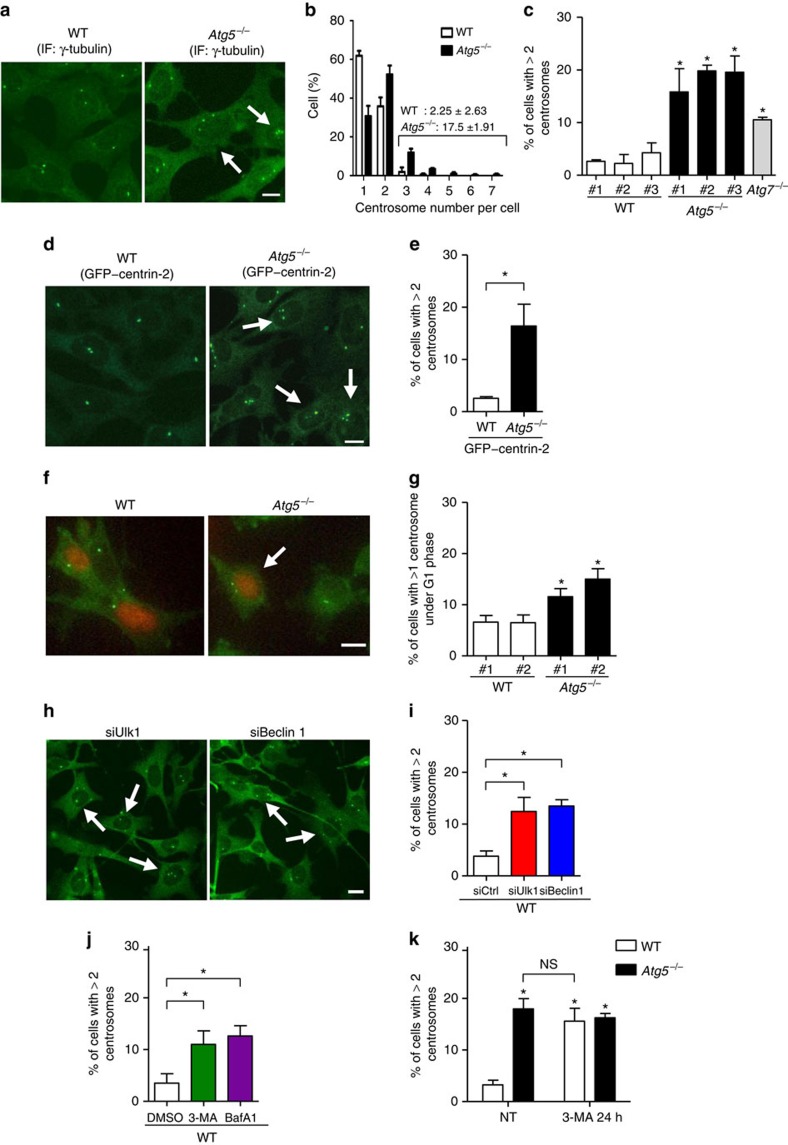
Suppression of autophagy increases centrosome number. (**a**) Centrosomes were immunostained with anti-γ-tubulin antibody in wild-type (WT) and *Atg5*^*−/−*^ MEFs. Arrows indicate cells with three or more centrosomes. (**b**) The percentage of cells with the number of centrosomes was obtained from the images of γ-tubulin staining. Open and closed columns indicate WT and *Atg5*^*−/−*^ MEFs, respectively (*n*=400 cells each). (**c**) The percentage of cells with three or more centrosomes. Indicated MEFs isolated from different embryos were immunostained with anti-γ-tubulin antibody and centrosomes per cell were counted. (**d**) The WT and *Atg5*^*−/−*^ MEFs were transfected with GFP–centrin-2. Arrows indicate cells with three or more centrosomes. (**e**) The percentage of WT and *Atg5*^*−/−*^ MEFs with three or more centrosomes was obtained from GFP–centrin-2-expressing cells. (**f**) The WT and *Atg5*^*−/−*^ MEFs were transfected with Fucci-orange and immunostained with anti-γ-tubulin antibody. The arrow indicates G1 cell with multiple centrosomes. (**g**) The percentage of cells with multiple centrosomes in the G1 phase was obtained. (**h**) The WT MEFs were transfected with siUlk1 and siBeclin-1 and were immunostained with anti-γ-tubulin antibody. Arrows indicate cells with three or more centrosomes. (**i**) The percentage of cells with three or more centrosomes is shown. (**j**) The WT MEFs were treated with 10 mM of 3-MA or 10 nM of bafilomycin A1 (BafA1) for 24 h and were immunostained with anti-γ-tubulin antibody. The percentage of cells with three or more centrosomes is shown. (**k**) The WT and *Atg5*^*−/−*^ MEFs were treated without (no treatment; NT) or with 10 mM of 3-MA for 24 h. The cells were immunostained with anti-γ-tubulin antibody and the percentage of cells with three or more centrosomes was calculated. Throughout, data are means+s.d. from three independent experiments and scale bar, 10 μm. In (**c**,**g**), *P<0.05 versus the value of WT#1 (analysis of variance (ANOVA) Tukey's *post hoc* test). In **e**, *P<0.05, Student's *t*-test. In **i** and **j**, *P<0.05, ANOVA Tukey's *post hoc* test. In **k**, *P<0.05 versus the value of WT NT (ANOVA Tukey's *post hoc* test). ‘NS' indicates no significant difference.

To determine whether autophagy is involved in centrosome number regulation, we first immunostained centrosomes with an anti-γ-tubulin antibody, a classical molecular marker of centrosomes, and counted the centrosomes in *Atg5*^*−/−*^ and control (wild-type) MEFs (Fig. 1a). Given that cells in the G1 and G2 phases contain one and two centrosomes, respectively^18^, cells with three or more centrosomes (γ-tubulin-positive dots) were considered as being defective in centrosome number regulation. The majority of wild-type MEFs contained one or two centrosomes; in contrast, ∼18% of the *Atg5*^*−/−*^ MEFs contained three or more centrosomes (Fig. 1a,b). Similar results were obtained in three other *Atg5*^*−/−*^ MEFs from different *Atg5*^*−/−*^ embryos and *Atg7*^*−/−*^ MEFs (Fig. 1c) and when centrosomes were counted using stably expressed green fluorescent protein (GFP)–centrin-2, which is a core component of centrioles^19^ (Fig. 1d,e). To further confirm centrosome number dysregulation in the *Atg5*^−/−^ MEFs, we focused on cells in the G1 phase. G1 cells can be visualized by the expression of Fucci-orange plasmids^20^, by which the nuclei of G1 cells are specifically labelled in orange (Fig. 1f). The population of cells with multiple centrosomes in the G1 phase was markedly larger in the *Atg5*^*−/−*^ MEFs than in the wild-type MEFs (Fig. 1f,g), confirming centrosome number dysregulation in the absence of autophagy. Consistent with the observation of multiple centrosomes, the number of cells with multipolar chromosome segregation was distinctly higher in the *Atg5*^*−/−*^ MEFs than in the wild-type MEFs (Supplementary Fig. 1). The possibility that the increase in centrosome number arose in the *Atg5*^*−/−*^ MEFs via a different cell cycle progression was rejected because the cell cycle had progressed equally in the wild-type and *Atg5*^*−/−*^ MEFs (Supplementary Fig. 2). All these data were obtained from SV40-transformed MEFs, and consistent results were obtained when primary MEFs (Supplementary Fig. 3) and primary hematopoietic cells (described later) were analysed.

To further confirm the involvement of autophagy in centrosome number regulation, we next evaluated the effects of other autophagy molecules and autophagy inhibitors using wild-type MEFs. The silencing of Ulk1 or Beclin 1 (Supplementary Fig. 4), which are autophagy-executing molecules^15^, increased the number of cells with multiple centrosomes (Fig. 1h,i). The addition of 3-methyladenine (3-MA) or bafilomycin A1, which are well-established autophagy inhibitors, yielded similar results (Fig. 1j). The population of cells with multiple centrosomes in 3-MA-treated wild-type MEFs was the same as that of the *Atg5*^*−/−*^ MEFs (Fig. 1k), confirming the contribution of autophagy to centrosome number regulation.

### The extra Cep63 dots are directly degraded by autophagy

**Figure 2:**
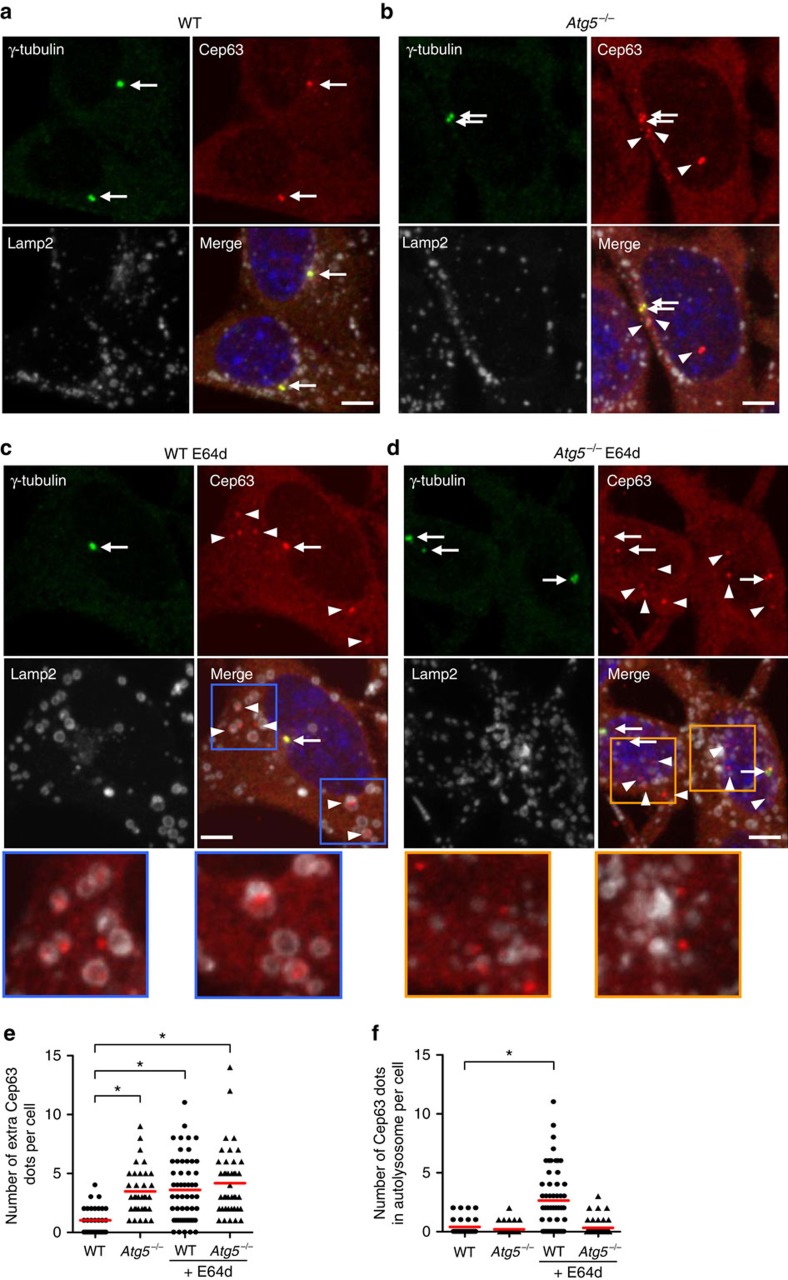
Regulation of extra Cep63 dots by autophagy. (**a**–**d**) The WT (**a**,**c**) and *Atg5*^*−/−*^ MEFs (**b**,**d**) were treated with (**c**,**d**) or without (**a**,**b**) 10 μg ml^−1^ of E64d. The cells were then immunostained with anti-γ-tubulin, anti-Cep63 and anti-Lamp2 antibodies and were examined by fluorescence microscopy. Representative images of γ-tubulin (green; upper left), Cep63 (red; upper right), Lamp2 (white; lower left) and the merged image (lower right) are shown. Arrows and arrowheads indicate mature centrosomes and extra Cep63 dots, respectively. Scale bar, 5 μm. Magnified images in the squares are shown below. Extra Cep63 dots increased in the *Atg5*^*−/−*^ MEFs and E64d-treated MEFs and were enclosed by Lamp2 vacuoles in the E64d-treated WT MEFs but not in the *Atg5*^*−/−*^ MEFs. (**e**) The number of extra Cep63 dots per cell was calculated (*n*>30 cells). The red lines indicate mean values. Asterisks indicate a significant difference (*P*<0.05, analysis of variance (ANOVA) Tukey's *post hoc* test). (**f**) The number of Cep63 dots enclosed by Lamp2 vacuoles was calculated (*n*>30 cells). The red lines indicate the mean value. Asterisk indicates a significant difference (*P*<0.05, ANOVA Tukey's *post hoc* test).

Identifying molecules involved in centrosome number dysregulation in the *Atg5*^*−/−*^ MEFs was a crucial issue to be addressed. We accordingly co-immunostained various centrosomal proteins together with γ-tubulin and compared their localization between the wild-type and *Atg5*^*−/−*^ MEFs. Because immunofluorescence signal intensity is easily altered by image acquisition and analysis, we fixed the experimental conditions and analysed the immunofluorescence without subjectivity using a Cell Imager. As a result, we observed characteristic immunofluorescence features of Cep63. Cep63 is a molecule that functions in the initial step of centriole duplication^6^,^21^,^22^. In the wild-type MEFs, Cep63 dots were mostly merged with γ-tubulin dots (Fig. 2a, arrows). In contrast, the several extra Cep63 dots (indicating Cep63 dots without the colocalization of γ-tubulin) were clearly observed in the *Atg5*^*−/−*^ MEFs (Fig. 2b, arrowheads and Fig. 2e). These extra Cep63 dots were also observed when immunofluorescence studies were performed using a different anti-Cep63 antibody (Supplementary Fig. 5). We next evaluated the possibility that the extra Cep63 dots are directly degraded by autophagy in the wild-type MEFs. The addition of E64d, a lysosomal protease inhibitor, allows the detection of constituents within autolysosomes by preventing autophagic degradation. When the wild-type MEFs were treated with E64d, the number of extra Cep63 dots increased to the equivalent level of the *Atg5*^*−/−*^ MEFs (Fig. 2c,e). Furthermore, simultaneous Lamp2 staining revealed that the extra Cep63 dots were mostly enclosed by Lamp2 vacuoles (autolysosomes; Fig. 2c, arrowheads; and Fig. 2f), suggesting that the extra Cep63 dots were engulfed and digested by autophagy in the absence of E64d. Because γ-tubulin-positive centrosomes were not enclosed by the Lamp2 vacuoles (Fig. 2c, arrow), the extra Cep63 dots, but not mature centrosomes, were the targets of autophagic degradation. In the *Atg5*^*−/−*^ MEFs, we observed several extra Cep63 dots, but none of them were enclosed by Lamp2 dots, because autolysosomes themselves were not generated by the lack of Atg5 (Fig. 2d,f). Consistent results were also obtained when extra Cep63 dots were examined using cells stably expressing GFP–Cep63, in which low levels of GFP–Cep63 was retrovirally expressed (Supplementary Fig. 6).

**Figure 3:**
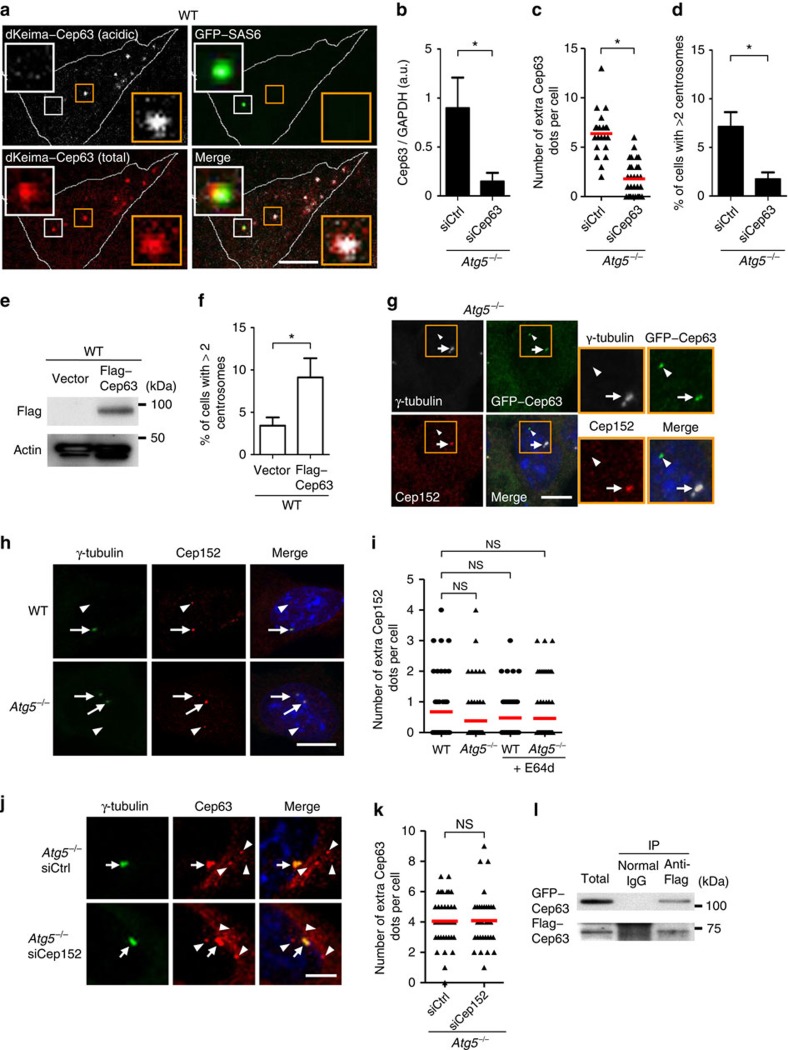
No colocalization of Cep152 with extra Cep63 dots. (**a**) Wild-type MEFs were transfected with dKeima–Cep63 and GFP–SAS6. Magnified images are shown at the upper left and lower right corners. White lines indicate the cell shape. (**b**–**d**) *Atg5*^*−/−*^ MEFs were transfected with siCep63 and scramble siRNA (siCtrl) for 24 h and were immunostained with anti-γ-tubulin antibody. (**b**) Quantitative PCR confirmed the reduction of *Cep63* mRNA. (**c**) The number of extra Cep63 dots per cell was calculated (*n*>30 cells). (**d**) The percentage of cells with three or more centrosomes is shown. (**e**,**f**) WT MEFs were transfected with Flag–Cep63 and a control vector for 24 h. (**e**) The expression of Flag–Cep63 was confirmed by western blotting. (**f**) The percentage of cells with three or more centrosomes is shown. (**g**) No colocalization of Cep152 with extra Cep63 dots in the *Atg5*^*−/−*^ MEFs. Arrow and arrowhead indicates mature centrosome and extra Cep63 dot, respectively. Magnified images are shown at the right. (**h**) No difference of extra Cep152 dots between WT MEFs and *Atg5*^*−/−*^ MEFs. Arrows and arrowheads indicate mature centrosomes and extra Cep152 dots, respectively. (**i**) The number of extra Cep152 dots per cell was calculated (*n*>30 cells). (**j**) No influence of Cep152 silencing on extra Cep63 dots. *Atg5*^*−/−*^ MEFs were transfected with siCep152 and siCtrl for 24 h. Arrows and arrowheads indicate mature centrosomes and extra Cep63 dots, respectively. Scale bar, 5 μm. (**k**) The number of extra Cep63 dots per cell was calculated (*n*>30 cells). (**l**) Lysates from MEFs expressing GFP–Cep63 and Flag–Cep63 were immunoprecipitated with anti-Flag antibody and normal IgG. ‘Total' indicates 5% of the lysates subjected to immunoprecipitation. In **e** and **l**, uncropped images are shown in Supplementary Fig. 14. Scale bar, 10 μm (**a**,**g**,**h**). In **b**,**d** and **f**, data are mean+s.d. (*n*=3). In **c**,**i** and **k**, the red lines indicate mean values. In **b**–**d** and **f**, *P<0.05, Student's *t*-test. In **i** and **k**, ‘NS' indicates no significant difference ((**i**) analysis of variance (ANOVA) Tukey's *post hoc* test, (**k**) Student's *t*-test).

The autophagic degradation of extra Cep63 dots was confirmed using dKeima^23^, a newly developed autophagy-sensitive probe. dKeima is a coral-derived fluorescent protein that emits different fluorescence signals in acidic and neutral environments, allowing the identification of proteins localizing in acid compartments such as autolysosomes. We expressed a dKeima–Cep63 fusion protein together with a GFP-fused SAS6 (ref. 24) protein in the wild-type MEFs and observed pH-dependent dKeima–Cep63 fluorescence. As shown in Fig. 3a, acidic dKeima–Cep63 dots, which indicated engulfed dots in autolysosomes, were not merged with GFP–SAS6 (yellow squares), whereas non-acidic dKeima–Cep63 dots were merged with the GFP fluorescence of GFP–SAS6 (white squares). Because GFP fluorescence can disappear as a result of quenching in acidic compartments, we also performed similar experiments by immunostaining for GFP–SAS6 using an anti-GFP antibody instead of the detection of GFP fluorescence, and obtained consistent results (Supplementary Fig. 7). These results confirmed that the extra Cep63 dots, but not SAS6-containing centrioles, were degraded in autolysosomes. Note that the presence of multiple dKeima–Cep63 dots was due to the transient and high expression level of *dKeima*–*cep63* via electroporetic transfection (Fig. 3a).

If Cep63 is a key molecule for the regulation of centrosome number, its expression level should influence the cell population with multiple centrosomes. Cep63 silencing in the *Atg5*^*−/−*^ MEFs (Fig. 3b) reduced the number of extra Cep63 dots (Fig. 3c) and also reduced the population of cells with multiple centrosomes (Fig. 3d; Supplementary Fig. 8). Although the number of *Atg5*^*−/−*^ MEFs with multiple centrosomes was decreased by transfection of short interfering RNA (siRNA; compare ‘siControl' in Fig. 3d with ‘*Atg5*^*−/−*^' in Fig. 1c), this may have been due to the influence of siRNA transfection. Consistent with siCep63 experiments, a high expression of *cep63* by electroporetic transfection in the wild-type MEFs increased the population of cells with multiple centrosomes (Fig. 3e,f; Supplementary Fig. 9), which is consistent with the previous reports^25^.

### Characterization of the extra Cep63 dots

What are the extra Cep63 dots? Because Cep152 is reported to interact with Cep63 to form a ring-like complex around the proximal end of centrioles^6^,^7^,^8^,^21^, we suspected the presence of Cep152 in Cep63 dots. However, the co-immunostaining analysis of GFP–Cep63 and Cep152 in *Atg5*^*−/−*^ MEFs unexpectedly revealed that Cep152 merged only with mature centrosomes containing γ-tubulin (Fig. 3g, arrow) and not with the extra Cep63 dots (Fig. 3g, arrowhead). The number of extra Cep152 dots per cell (<0.5 dots per cell) was far less than that of extra Cep63 dots (∼4 dots per cell) in *Atg5*^*−/−*^ MEFs (Fig. 3h,i). No difference was observed in Cep152 immunofluorescent dots between the wild-type and *Atg5*^*−/−*^ MEFs (Fig. 3h,i). Furthermore, the silencing of Cep152 did not alter the number and size of the extra Cep63 dots in the *Atg5*^*−/−*^ MEFs (Fig. 3j,k). Collectively, these results indicated that Cep152 is not recruited to the extra Cep63 dots. Because Cep152 is required for the proper recruitment of Plk4 and SAS6 (ref. 26), it is reasonable that the extra Cep63 dots fail to become mature centrosomes (Fig. 3g), consistent with the results shown in Fig. 2 and Fig. 3a. We observed no colocalization of centriolar coiled-coil protein 110 kDa (CP110), CPAP and centrin-2 with the extra Cep63 dots (Supplementary Fig. 10). SAS6 also did not merge with the extra Cep63 dots, as shown above (Fig. 3a). We observed no differences in the immunofluorescence dots of Cep135, CP110 and CPAP between the wild-type and *Atg5*^*−/−*^ MEFs (Supplementary Fig. 11). Thus, the extra Cep63 dots did not include these molecules required for centrosomal biogenesis. Because we did not examine all centrosomal proteins, the presence of other centrosomal proteins that were not analysed cannot be excluded. Unlike the other proteins, we found the self-association of Cep63, because an interaction between GFP–Cep63 and Flag–Cep63 was observed (Fig. 3l). Thus, the extra Cep63 dots may have been generated by Cep63 homo-oligomerization.

Where were the extra Cep63 dots generated? Because the extra Cep63 dots were spread throughout the cytoplasm, we first hypothesized that the extra Cep63 dots had been generated *de novo*. However, when microtubules were interfered by nocodazole, the extra Cep63 dots were located only surrounding the centrosomes (Supplementary Fig. 12), suggesting that the Cep63 dots were generated close to the mother centriole and that they move along microtubules.

### Involvement of p62 in autophagic Cep63 regulation

**Figure 4:**
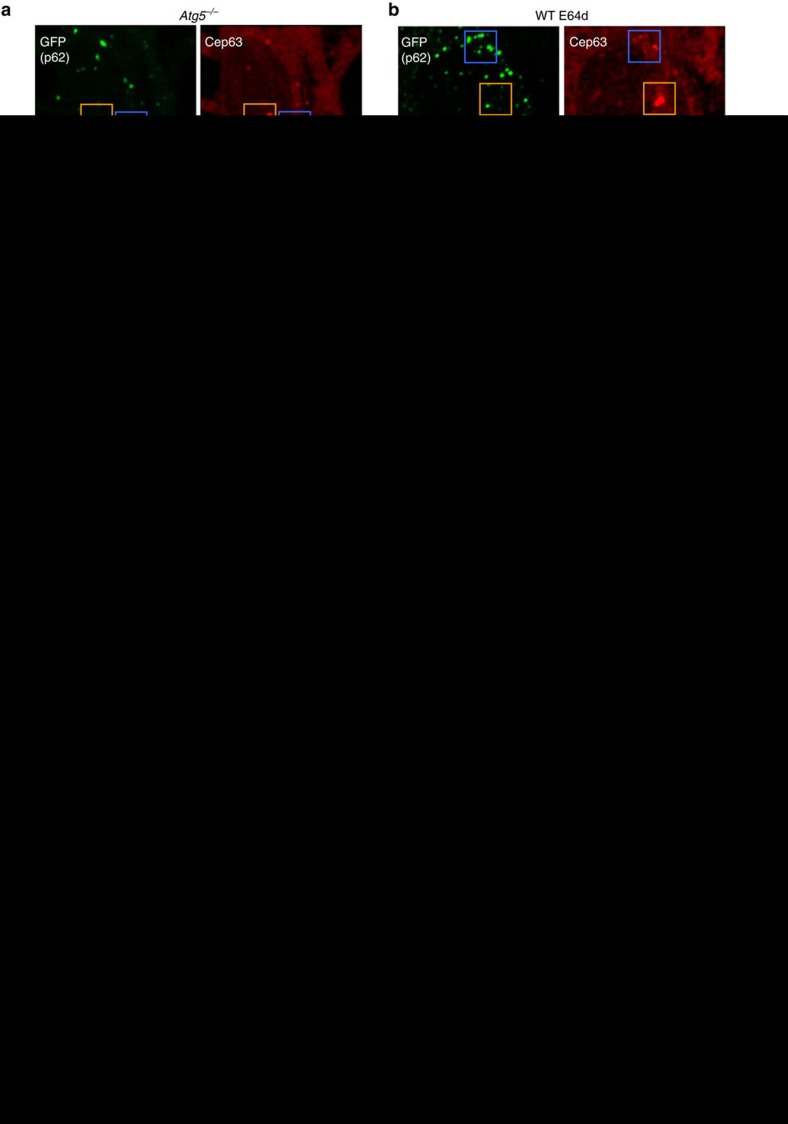
Interaction between Cep63 and p62. (**a**–**c**) The colocalization of p62 and Cep63 in extra Cep63 dots, but not mature centrosomes. (**a**) *Atg5*^*−/−*^ MEFs transfected with GFP–p62 were immunostained with anti-GFP, anti-Cep63 and anti-γ-tubulin antibodies. The mature centrosome containing γ-tubulin did not merge with p62 (orange squares), whereas extra Cep63 dots without the presence of γ-tubulin merged with p62 (blue squares). The magnified images of each structure are shown at the bottom. Arrowheads indicate Cep63 dots colocalizing with p62. Arrow indicates mature centrosome without colocalizing p62. (**b**) The same experiment was performed using E64d-treated WT MEFs, instead of *Atg5*^*−/−*^ MEFs. (**c**) The number of Cep63 dots colocalizing with p62 was calculated (*n*>25 cells). Red lines indicate mean values. (**d**–**f**) The interaction of p62 with Cep63. Lysates from GFP–Cep63-expressing MEFs (**d**) and Flag(3 × )–Cep63-expressing MEFs (**e**) were immunoprecipitated with anti-p62 antibody and normal IgG. In **f**, *in vitro*-translated p62 and *in vitro*-translated Flag–Cep63 proteins were incubated and also immunoprecipitated with anti-p62 antibody and normal IgG. The immune complexes were then analysed by western blotting using anti-GFP, anti-Flag and anti-p62 antibodies. ‘Total' indicates 5% of the lysates subjected to immunoprecipitation. Uncropped images are shown in Supplementary Fig. 14. (**g**,**h**) Close proximity assay between Cep63 and p62. (**g**) The WT, *Atg5*^*−/−*^ and *p62*^*−/−*^ MEFs were transiently transfected with Flag(3 × )–Cep63 for 24 h, and the Cep63–p62 interaction was visualized using anti-Flag and anti-p62 antibodies with a Duolink detection kit. Red signals indicate positive interaction. In the lower right panel, a quantitative analysis of signal intensity is shown. The signal area per cell was calculated from >50 cells using In Cell Analyzer. Data are shown as proportions of the value of WT (mean+s.d.). (**h**) Experiments similar to that shown in **g** were performed using the *p62*^*−/−*^ MEFs. Scale bar, 5 μm (**a**,**b**) and 10 μm (**g**,**h**). In (**c**,**g**,**h**), *P<0.05, analysis of variance Tukey's *post hoc* test. ‘NS' indicates no significant difference (*n*=3).

How are the Cep63 dots degraded by autophagy? To address this question, we investigated the involvement of p62, given that this protein functions as an adaptor or cargo receptor for autophagic degradation^13^. If Cep63 binds to p62 as an autophagy substrate, Cep63 is expected to colocalize with p62. When we immunostained Cep63 and γ-tubulin in GFP–p62 expressing *Atg5*^*−/−*^ MEFs, we observed several colocalization dots of p62 and γ-tubulin-negative Cep63 (Fig. 4a; blue squares, arrowheads), but not of γ-tubulin-positive Cep63 (Fig. 4a; orange squares, arrow). We observed similar results in E64d-treated wild-type MEFs (Fig. 4b). These observations supported an inference that mature centrosomes are not engulfed and digested. Quantitative analysis revealed that the number of Cep63–p62 colocalizing dots increased in cells lacking autophagy (Fig. 4c). These results suggested the involvement of p62 in the autophagic degradation of Cep63.

To elucidate the physical interaction between p62 and Cep63, we performed a co-immunoprecipitation assay with anti-p62 antibody in cells expressing GFP–Cep63 or Flag–Cep63, given that no anti-Cep63 antibody for western blotting was available. As shown in Fig. 4d,e, both GFP–Cep63 and Flag–Cep63 were efficiently co-immunoprecipitated by the anti-p62 antibody. Consistently, *in vitro*-translated p62 bound efficiently to *in vitro*-translated Flag–Cep63 (Fig. 4f), indicating an interaction between p62 and Cep63. We further visualized this interaction by employing a close proximity assay. This assay allows the generation of an interaction signal using antibodies against molecules of interest. Positive signals can be observed when the distance between two molecules is <30–40 nm. When we investigated the interaction between Cep63 and p62 in the wild-type MEFs, we detected several positive signals (Fig. 4g: WT). These interaction signals were stronger in the *Atg5*^*−/−*^ MEFs than in the wild-type MEFs (Fig. 4g: *Atg5*^*−/−*^), which was consistent with the colocalization analysis results (Fig. 4c). A strong reduction of interaction signals in the *p62*^*−/−*^ MEFs (Fig. 4g) and its reversal by the introduction of full-length p62 (Fig. 4h) validated this procedure and confirmed the interaction between Cep63 and p62.

**Figure 5:**
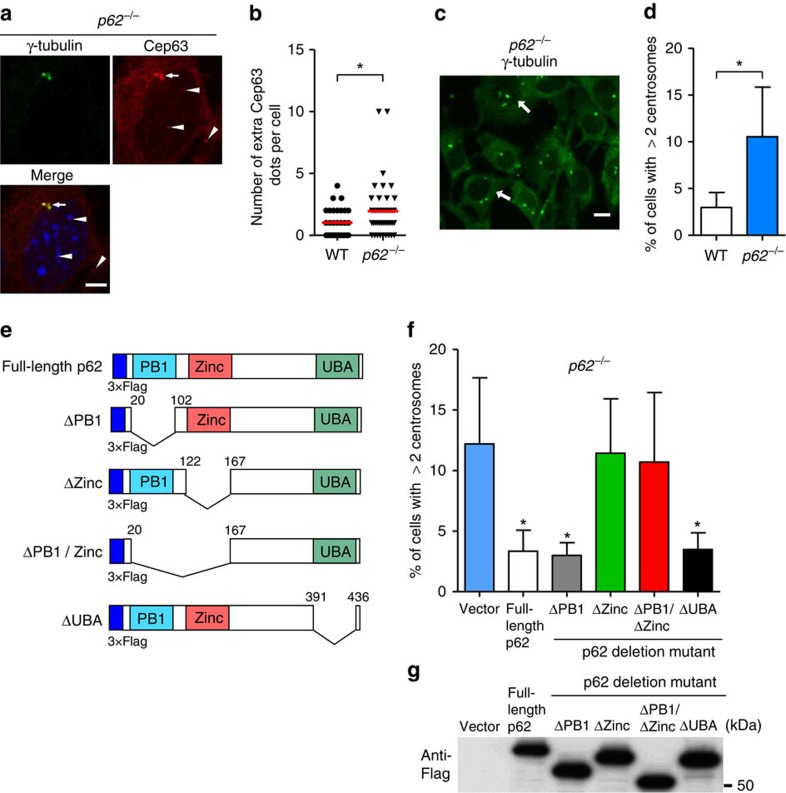
Involvement of p62 in centrosome number regulation. (**a**,**b**) Increase in extra Cep63 dots in *p62*^*−/−*^ MEFs. *p62*^*−/−*^ MEFs were immunostained with anti-γ-tubulin and anti-Cep63 antibodies and were examined by fluorescence microscopy. Representative images of γ-tubulin (green), Cep63 (red) and the merged image are shown in **a**. The nucleus was stained with 4,6-diamidino-2-phenylindole (DAPI) (blue). Arrow and arrowheads indicate mature centrosome and extra Cep63 dots, respectively. Scale bar, 5 μm. (**b**) The number of extra Cep63 dots per cell was calculated (*n*>30 cells). The red lines indicate mean values. The asterisk indicates a significant difference (*P*<0.05, Student's *t*-test). (**c**,**d**) Increase in mature centrosomes in the *p62*^*−/−*^ MEFs. The *p62*^*−/−*^ MEFs were immunostained with anti-γ-tubulin antibody and were examined by fluorescence microscopy. A representative image is shown in **c**. Arrows indicate cells with multiple centrosomes. Scale bar, 10 μm. (**d**) The percentage of cells with three or more centrosomes is shown as the mean+s.d. (*n*=3). The asterisk indicates a significant difference (*P*<0.05, Student's *t*-test). (**e**) The design of p62 mutant plasmids. The p62 protein contains PB1, zinc-binding and UBA domains. Each deletion plasmid was generated. (**f**) The *p62*^*−/−*^ MEFs were transfected with each Flag(3 × )-tagged plasmid and were immunostained with anti-γ-tubulin antibody. The percentage of cells with three or more centrosomes was calculated. Data are mean+s.d. (*n*=3). *P<0.05 versus the value of vector (analysis of variance (ANOVA) Tukey's *post hoc* test). (**g**) The equal expression of each p62 mutant was confirmed by western blotting using anti-Flag antibody. Uncropped images are shown in Supplementary Fig. 14.

If p62 is involved in the regulation of centrosome number, the number of cells with extra Cep63 dots and multiple centrosomes is expected to increase in the *p62*^*−/−*^ MEFs. The *p62*^*−/−*^ MEFs displayed extra Cep63 dots (Fig. 5a, arrowheads; and Fig. 5b) and more centrosomes (Fig. 5c,d), similar to the *Atg5*^*−/−*^ MEFs, although the effect was less pronounced than that observed in the *Atg5*^*−/−*^ MEFs. A reason for this is the compensation of the loss of p62 by Nbr1, which is a homologue of p62. In fact, the silencing of Nbr1 largely increased the number of cells with multiple centrosomes in *p62*^*−/−*^ MEFs, but not in normal MEFs (Supplementary Fig. 13). An alternative possibility is that the lack of p62 reduced the efficiency of substrate recognition but not autophagy itself. The involvement of p62 was further confirmed by the reduction of centrosome number following full-length p62 overexpression in the *p62*^*−/−*^ MEFs (Fig. 5e,f). p62 contains several protein interaction domains with structural motifs^27^, including an Ub-associated (UBA) domain, a zinc finger domain interacting with RIP, and a PB1 domain that binds PKC and ERK (Fig. 5e). Accordingly, we attempted to determine the regions of p62 involved in centrosome number regulation by introducing p62 deletion mutant plasmids into the *p62*^*−/−*^ MEFs. As shown in Fig. 5f, p62 ΔPB1 and ΔUBA mutants rescued the number of centrosomes in the same way as full-length p62 plasmid expression, whereas the p62 Δzinc finger mutant did not affect centrosome number even though the expression levels of the p62 mutants were comparable (Fig. 5g). Consistently, Cep63–p62 Duolink interaction signals were not rescued by the introduction of a p62 Δzinc finger mutant into the *p62*^*−/−*^ MEFs (Fig. 4h). We conclude that the zinc finger domain of p62 is crucial for the regulation of centrosome number.

### Autophagy-dependent centrosome number regulation *in vivo*

**Figure 6:**
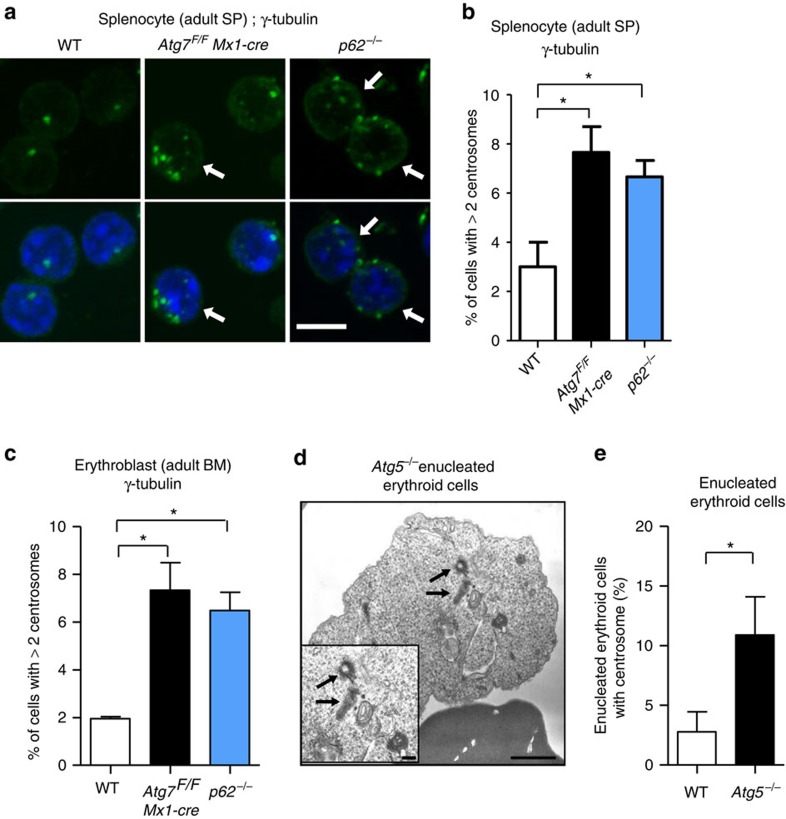
Increase in the number of centrosomes in autophagy-deficient and *p62*^*−/−*^ mice. (**a**,**b**) Increase in the number of centrosomes in autophagy-deficient and *p62*^*−/−*^ mice. Splenocytes were isolated from the indicated mice at the age of 20 weeks, and centrosomes were stained with anti-γ-tubulin antibody. Representative images are shown in **a**. Centrosomes were stained green. The nucleus was stained with 4,6-diamidino-2-phenylindole (DAPI) (blue). Arrows indicate cells with extra centrosomes. Scale bar, 5 μm. (**b**) The percentage of cells with three or more centrosomes was calculated. Data are expressed as the mean+s.d. (*n*=3). Asterisks indicate a significant difference (*P*<0.05, analysis of variance (ANOVA)). (**c**) Bone marrow erythroblasts were isolated from the indicated mice at the age of 20 weeks, and centrosomes were stained with an anti-γ-tubulin antibody. Then, the percentage of cells with three or more centrosomes was calculated. Data are expressed as the mean+s.d. (*n*=3). Asterisks indicate a significant difference (*P*<0.05, ANOVA). Note that the erythroblasts were isolated at 2 weeks after PIPC injection in the experiments of Atg7 cKO mice, when Atg7 was almost completely deleted from hematopoietic cells. (**d**,**e**) Enucleated erythroid cells from the indicated mouse embryos at embryonic day 14.5 were analysed by electron microscopy (EM). A representative EM image of an *Atg5*^*−/−*^ erythrocyte is shown in **d**. Centrioles are clearly observed (arrows). Scale bar, 1 μm. A magnified image is shown in the inset. Scale bar, 0.1 μm. (**e**) The percentages of enucleated erythroid cells with centrosomes were calculated from the EM images (*n*=50 cells each). Data are shown as the mean+s.d. (*n*=3). The asterisk indicates a significant difference (*P*<0.05, Student's *t*-test).

We investigated whether centrosome number increased *in vivo*. We examined centrosome number in splenic cells and bone marrow erythroblasts derived from polyinosine–polycytidine-injected *Atg7*^*F/F*^*: Mx1-cre* mice^28^ (hereafter described Atg7 cKO mice). In Mx1-cre mice, the Cre recombinase is under the control of the *Mx1* promoter, and is induced by the administration of polyinosine–polycytidine, which abolishes Atg7 from hematopoietic cells, hepatocytes, splenocytes and so on. When we counted the centrosomes per cell by immunostaining centrosomes with an anti-γ-tubulin antibody, we found a greater number of splenocytes with multiple centrosomes in the Atg7 cKO mice than that in wild-type mice (Fig. 6a,b). Similar results were observed in splenocytes from *p62*^*−/−*^ mice^29^ (Fig. 6a,b) and bone marrow erythroblasts from the Atg7 cKO mice and *p62*^*−/−*^ mice (Fig. 6c). Although enucleated erythroid cells do not naturally have centrosomes, we observed one or two centrosomes in some *Atg5*^*−/−*^ enucleated erythroid cells (Fig. 6d,e), indicating that Atg5-dependent autophagy is involved in centrosome clearance in erythrocytes. Thus, centrosome number is regulated by autophagy *in vivo*.

Taken together, all the data indicated that autophagy degrades Cep63 via interaction with p62 for maintaining proper centrosome number.

## Discussion

We have shown that autophagy participates in centrosome number regulation. The Ub–proteasome system is one of the mechanisms contributing to the absence of the production of extra centrosomes. The SKP1/Cullin/F-box (SCF)^Slimb^ Ub ligase reportedly regulates centrosome overduplication via the regulation of Plk4 expression levels^10^,^11^. The BRCA1-dependent ubiquitination of γ-tubulin also regulates centrosome number^30^. We here show that in addition to the Ub–proteasome system, selective autophagy is another protein degradation system that participates in centrosome number regulation. Both selective autophagy and the Ub–proteasome system may digest different specific molecules for maintaining proper centrosome numbers. Ciliogenesis is also regulated by these two protein degradation systems^31^,^32^,^33^.

How does autophagy control centrosome number? We first hypothesized that mature centrosomes are digested by autophagy when excess centrosomes are present. However, mature centrosomes did not interact with p62 (Fig. 4) and were not engulfed in autolysosomes (Fig. 2). We accordingly concluded that mature centrosomes are not the target substrate of autophagy. Instead, we found that several Cep63 dots were generated in autophagy-deficient cells and that these dots were directly associated with p62 and were engulfed into autolysosomes in autophagy-driving cells, indicating the role of Cep63 in the autophagic regulation of centrosome number. Given that Cep63 functions at the proximal end of the mother centriole^21^,^22^ and that many Cep63 dots were observed close to mature centrosomes in the *Atg5*^*−/−*^ MEFs upon nocodazole treatment (Supplementary Fig. 12), many Cep63 dots are expected to be generated near the mother centriole. In autophagy-performing cells, almost all these Cep63 dots are rapidly degraded by autophagy to regulate centrosome number. In contrast, in autophagy-deficient cells, many Cep63 dots remain and interact with p62, but not Cep152; these are the ‘extra Cep63 dots'. In addition, a few Cep63 dots are thought to interact with Cep152, instead of p62, to eventually become a mature centrosome, which results in the generation of extra centrosomes.

Recently, Zhao *et al*.^34^ reported that Cep63 interacts with UVRAG, and hence UVRAG is capable of regulating centrosome number. The disruption of this interaction causes an increase in centrosome number. Although UVRAG functions to regulate autophagy maturation (autophagosome–lysosome fusion)^16^, UVRAG-dependent centrosomal regulation was not mediated by autophagy, because UVRAG-dependent centrosome regulation can be observed even in *Atg5*-deficient cells^34^. Importantly, the increase in centrosome number in *Atg5*^*−/−*^ MEFs was also observed in their data, although they did not point this out. Thus, Cep63 appears to be regulated by an independent dual system, involving an autophagy-dependent and UVRAG-dependent mechanism. In conclusion, our findings have shown a novel role for autophagy in centrosome number regulation.

## Methods

### Antibodies and chemicals

Anti-γ-tubulin (T6557; 1:500) and anti-FLAG (M2; F1804; WB: 1:1,000, IF: 1:500) mouse monoclonal antibodies were purchased from Sigma-Aldrich. Anti-α-tubulin (A11126; 1:100) mouse monoclonal antibody was from Invitrogen. Anti-GFP (GF090R; 1:200), anti-Lamp2 (GL2A7; 1:200) and anti-Ter119 (#553670; 1:200) rat monoclonal antibodies were obtained from Nacalai Tesque, Abcam and BD Biosciences, respectively. Anti-CEP135 (ab75005; 1:100), anti-Ulk1 (A7481; 1:1,000) and anti-Cep63 (06-1292; 1:200) polyclonal antibodies were purchased from Abcam, Sigma-Aldrich and Millipore, respectively. Anti-Cep152 (A302-480A; 1:200) and anti-CP110 (A301-343A; 1:200) polyclonal antibodies were from Bethyl. Anti-Beclin1 (PD017; 1:1,000) and anti-p62/SQSTM1 (PM045; WB: 1:5,000, IF: 1:500) polyclonal antibodies were from Medical & Biological Laboratories (MBL). Anti-CPAP (11517-1-AP; 1:200) and anti-CEP63 (16268-1-AP; 1:200) polyclonal antibodies were from Proteintech Group. 3-MA, Bafilomycin A1 and E64d were from Sigma-Aldrich, and other chemicals were purchased from Nacalai Tesque.

### Animals

The generation of the *p62*^*−/−*^, *Atg5*^*−/−*^ and Atg7-flox mice have been described elsewhere^28^,^29^,^35^. The Mx1-cre mice were purchased from The Jackson Laboratory. The Atg7^F/F^; Mx1-cre mice were generated by crossbreeding. The expression of Cre was induced by five intraperitoneal injections of 300 μg of polyinosinic-polycytidylic acid (PIPC). All mice were maintained in a specific pathogen-free animal facility at the Laboratory for Recombinant Animals (Medical Research Institute, Tokyo Medical and Dental University, Tokyo, Japan). All experiments were reviewed and approved by the Institutional Animal Care and Use Committee in Tokyo Medical and Dental University, and were conducted according to the committees' guidelines.

### Cell culture and DNA transfection

MEFs were generated from the wild-type, *Atg5*^*−/−*^ and *p62*^*−/−*^ embryos at embryonic day 13.5 and were immortalized with SV40 T antigen. Wild-type and *p62*^*−/−*^ primary splenocytes were collected as described previously^36^. These cells were cultured in Dulbecco's modified Eagle's medium supplemented with 2 mM of L-glutamine, 1 mM of sodium pyruvate, 0.1 mM of non-essential amino acids, 10 mM of HEPES/Na^+^, (pH 7.4), 0.05 mM of 2-mercaptoethanol, 100 U ml^−1^ of penicillin, 100 μg ml^−1^ of streptomycin and 10% of fetal bovine serum.

DNAs encoding mouse centrin-2, mouse SAS6 and human Cep63 were cloned into a pMSCV–GFP–Zeo vector. Human *cep63* was also cloned into a dKeima/pCS2 vector provided by RIKEN and into a pMSCV–Flag(3 × ) vector. The Fucci-orange plasmid was also provided by RIKEN. Mouse *p62* mutant DNAs (ΔPB1 (21–102a.a), ΔZinc (123–167 a.a), ΔPB1/Zinc1 (21–102 and 123–167 a.a) and ΔUBA (392–436 a.a)) were generated by PCR and were subcloned into the pMSCV–Flag(3 × ) vector. Each GFP fusion plasmid was introduced into MEFs by retroviral infection using Plat-E cells. Other plasmids were transfected into MEFs using the neon electroporation system. The transfection efficiency was >75%, as assessed by co-transfection with DNA expressing GFP. In the study using siRNA, cells (1 × 10^6^) were transfected with 10 μg of siRNA by lipofection using Lipofectamine RNAiMAX. The siRNA sequences used were as follows: mouse Ulk1, 5′-GGGUGGACACAUGCUAAUA-3′; mouse Beclin1, 5′-GGUUUGGAAAGAUGCUUUA-3′; mouse Cep63, 5′-GCUGGAAUCUCUCAAAUUA-3′; mouse Cep152, 5′-GUCAUUAAUUACUAUUUAA-3′. Control siRNA (Dharmacon siGENOME Non-Targeting siRNA#1 D-001210-01-20; Thermo Scientific, Rockford, IL, USA) was also used.

### Immunofluorescence analysis

Cells were washed twice with phosphate-buffered saline (PBS), fixed in 99% cold methanol and incubated with 1% bovine serum albumin (BSA) plus 0.05% Triton X-100 at room temperature for 30 min. The primary antibody in 1% BSA was then added for ∼12 h at 4 °C. After washing twice, the cells were treated with the corresponding secondary antibody conjugated with Alexafluor488, 568 or 633 and were examined under a confocal microscope (Zeiss LSM710).

In the study of GFP–centrin-2, MEFs stably expressing GFP–centrin-2 were cultured on glass coverslips and were fixed in cold (−20 °C) methanol for at least 5 min, rehydrated in PBS, and examined under a fluorescence microscope (Olympus). In some experiments, the cells were analysed with imaging analyser (In Cell Analyser 3000, GE Healthcare Co.).

For dKeima assay, dKeima and GFP fluorescence was observed in living cells and then fixed with 4% PFA. Fixed cells were stained with anti-GFP antibody and observed same cell that analysed dKeima fluorescence.

### Protein interaction assay

For the production of recombinant proteins, mouse *p62* and human *Cep63* were cloned into a pBSK–Flag(3 × ) vector. Flag–Cep63 and p62 were *in vitro* translated using TNT Quick Coupled System (Promega). For the immunoprecipitation assay, cell extracts were prepared from MEFs transfected with Flag–Cep63 or GFP–Cep63. Lysates or *in vitro*-translated proteins were then incubated with anti-p62 antibody and normal rabbit IgG as a negative control for 2 h at 4 °C. Immune complexes were captured with Dynabeads protein G (Invitrogen) and were washed four times. The co-immunoprecipitation of Cep63 was detected by Western blotting using anti-Flag or anti-GFP antibody.

For the close proximity assay, a Duolink proximity ligation assay was performed according to the manufacturer's instructions. Briefly, Flag(3 × )–Cep63-expressing cells were grown on coverslips and were fixed in 99% cold methanol for 15 min. The cells were then permeabilized in PBS containing 0.5% NP-40 for 15 min. After 30 min blocking in PBS with 0.2% coldwater fish gelatin and 0.5% BSA, primary antibodies were applied. After washing the cells, proximity ligation assay probes were added, which was followed by hybridization, ligation and amplification at 37 °C. Fluorescence images were then acquired and were quantitatively analysed with the In Cell Analyser 2000 (GE Healthcare).

### Electron microscopy

Cells were fixed by a conventional method (1.5% paraformaldehyde/3% glutaraldehyde in 0.1 M of phosphate buffer at pH 7.2, followed by an aqueous solution of 1% osmium tetroxide). The fixed cells were embedded in Epon 812, and thin sections (70–80 nm) were cut and stained with uranyl acetate^17^ and lead citrate for observation under a JEOL-1010 instrument (JEOL, Tokyo, Japan) at 80 kV.

### Statistical analysis

Results are expressed as the mean+s.d. Statistical evaluation was performed using Prism (GraphPad) software. The comparisons of two data sets were performed by unpaired two-tailed Student's *t*-test. All other comparisons of multiple data sets were performed using one-way analysis of variance followed by Tukey's *post hoc* test. Statistical significance was declared for *P* values of<0.05. Statistical analyses of nonrandom associations between two categorical variables were examined using Fisher's exact test.

### Data availability

The authors declare that all data supporting the findings of this study are available within the paper and its Supplementary Information files or are available from the corresponding author on request.

## Additional information

**How to cite this article:** Watanabe, Y. *et al*. Autophagy controls centrosome number by degrading Cep63. *Nat. Commun.* **7,** 13508 doi: 10.1038/ncomms13508 (2016).

**Publisher's note:** Springer Nature remains neutral with regard to jurisdictional claims in published maps and institutional affiliations.

## Supplementary Material

Supplementary Information Supplementary Figures 1-14
