# Supplementary material for: Autophagy controls centrosome number by degrading Cep63
Source: Nat Commun. 2016 Nov 21;7:13508. doi: 10.1038/ncomms13508 (PMC5473638; doi:10.1038/ncomms13508)
Supplement: Supplementary Information — Supplementary Figures 1-14 [file ncomms13508-s1.pdf]

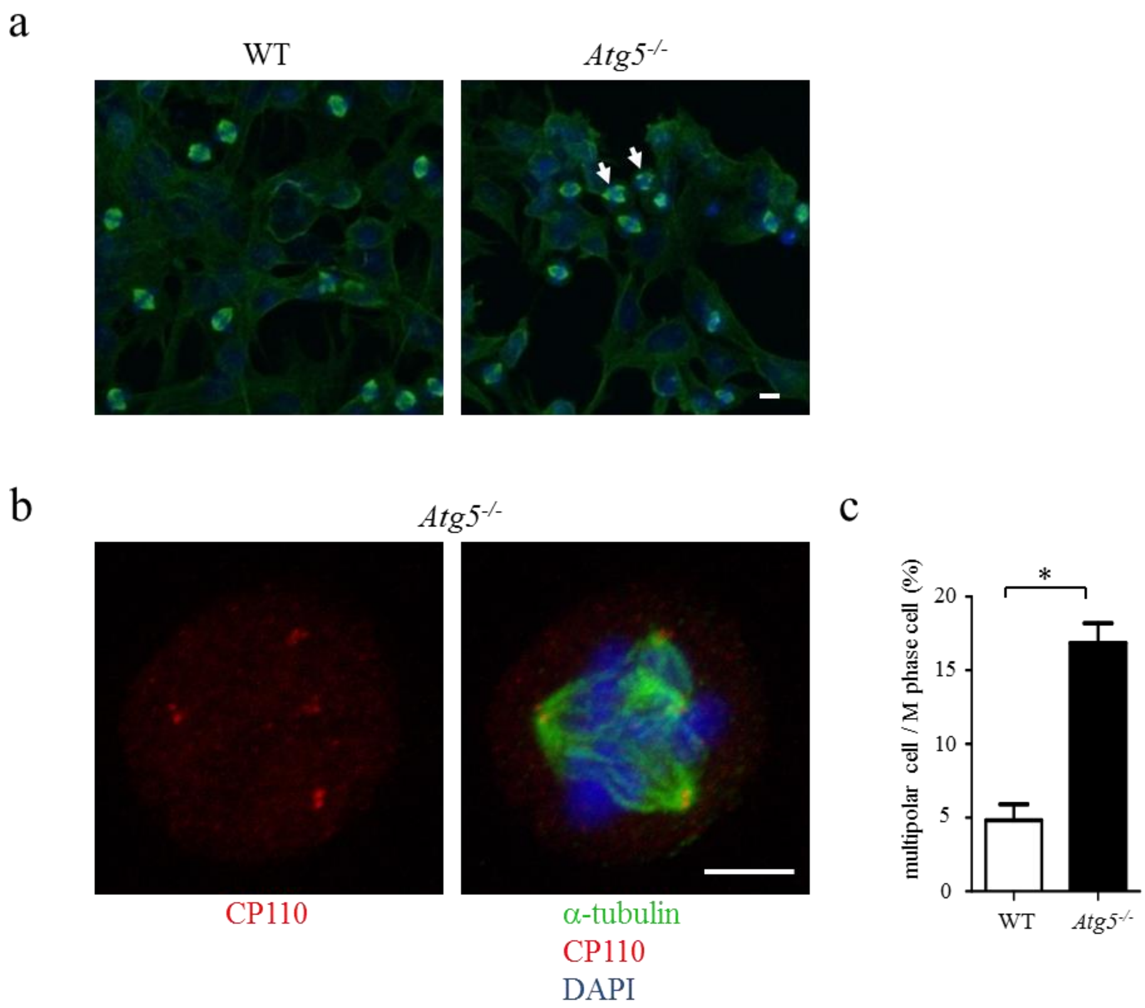

Supplementary Figure 1 Increase in multipolar cells in *Atg5*$^{-/-}$ MEFs.

(a) WT and *Atg5*$^{-/-}$ MEFs were synchronized at the G1/S boundary with a thymidine block. Briefly, the cells were treated with 2 mM of thymidine for 24 h, washed twice with PBS, grown for 3 h in standard medium, and treated with 20 μM of MG132 for 3 h to arrest the cell cycle at the M-phase. The cells were then immunostained with anti-α-tubulin antibody (green) to show spindle fibers. DNA was also visualized with DAPI (blue). Arrows indicate multipolar cells. Scale bar = 10 μm. **(b)** Representative images of multipolar *Atg5*$^{-/-}$ MEFs. The cells were immunostained with anti-α-tubulin (green) and anti-CP110 (red) antibodies. DNA was visualized with DAPI (blue). Scale bar = 5 μm. **(c)** The number of multipolar cells per M-phase cell was calculated (n > 50 cells). Data are shown as mean + s.d. (n=3). The asterisk indicates a significant difference ($p < 0.05$, Student's *t*-test).

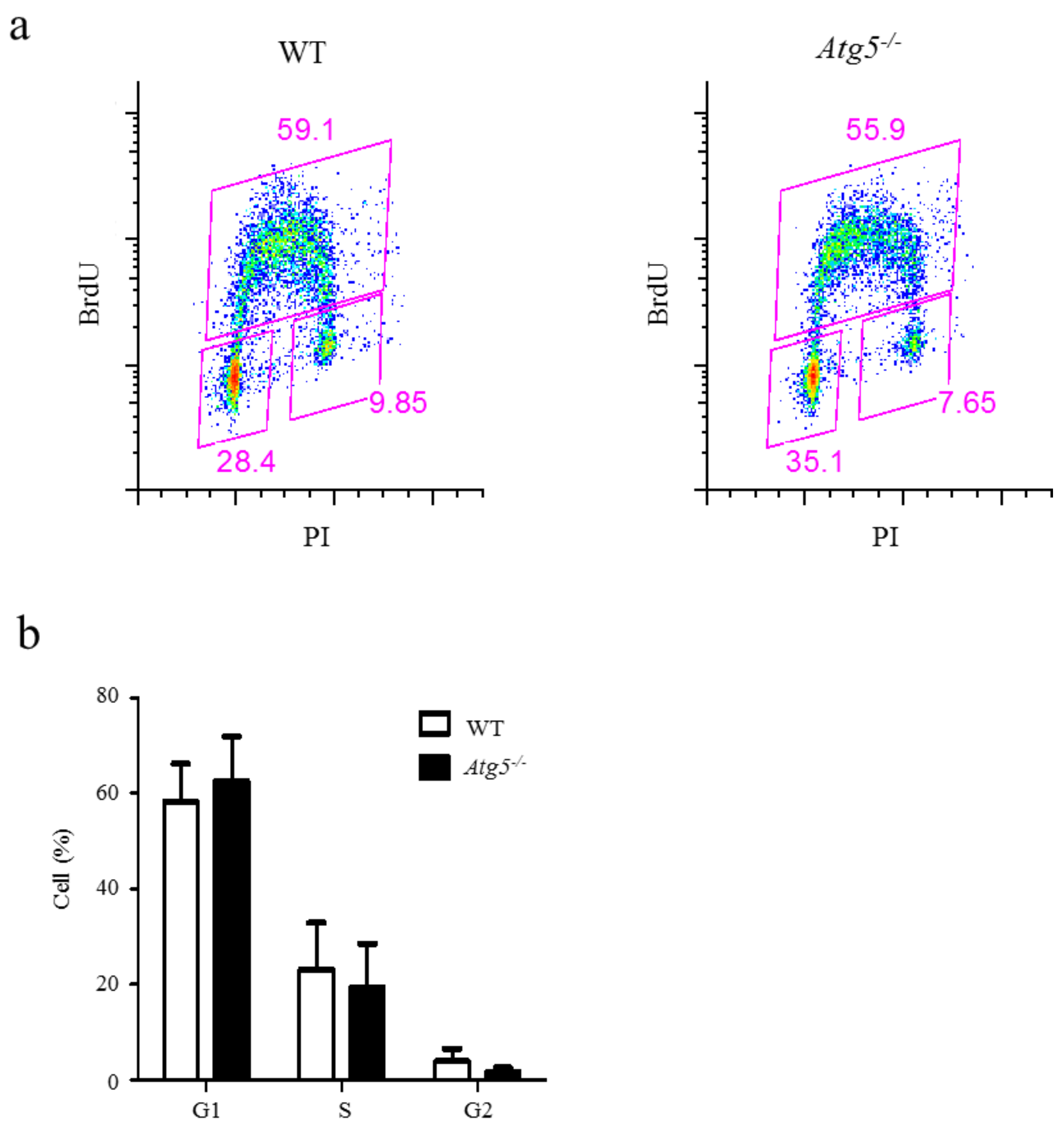

Supplementary Figure 2 Equivalent progression of cell cycle between wild-type MEFs and *Atg5*$^{-/-}$ MEFs.

(a) WT and *Atg5*$^{-/-}$ MEFs were stained with BrdU for 2 h. After PI staining, the cells were analyzed by flow cytometry. Representative cell cycle analyses are shown. BrdU^{low}PI^{low}, BrdU^{high}, and BrdU^{low}PI^{high} fractions indicate the G1, S and G2/M phases, respectively. The numbers indicate the population size of each fraction. **(b)** The population size of cells in each cell-cycle fraction was obtained by flow cytometric analysis. Open and closed columns indicate the WT and *Atg5*$^{-/-}$ MEFs, respectively. Data are shown as mean + s.d. (n=3).

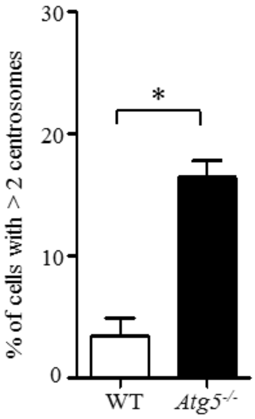

Supplementary Figure 3 Increase in centrosome number in primary *Atg5*$^{-/-}$ MEFs.

Centrosomes were immunostained with an anti-γ-tubulin antibody in primary wild-type (open column) and primary *Atg5*$^{-/-}$ MEFs (closed column). The percentage of cells with more than three or more centrosomes was obtained. Data are the mean + s.d. (n = 3). The asterisk indicates a significant difference ($p < 0.05$, Student's *t*-test).

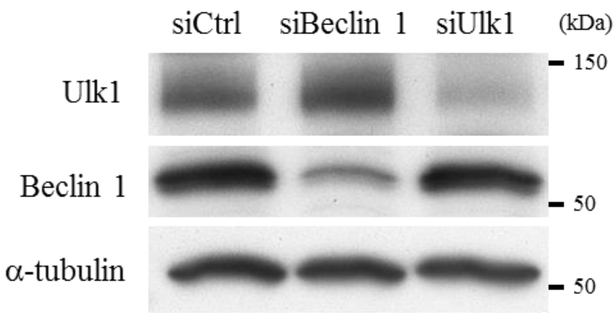

Supplementary Figure 4 Efficient suppression of Ulk1 and Beclin 1.

Wild-type MEFs were transfected with Ulk1 siRNA, Beclin 1 siRNA, and control siRNA. After 24 h, the cells were lysed, and the expression levels of Ulk1 and Beclin 1 were determined by Western blot analysis. “α-tubulin” is a loading control. Uncropped images are shown in Supplementary Fig. 14.

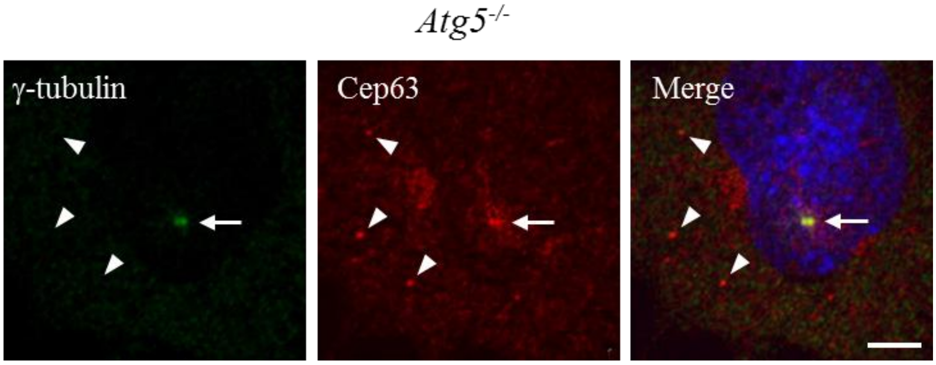

Supplementary Figure 5 Regulation of extra Cep63 dots by autophagy.

The same experiment as shown in Figure 2b was performed using a different anti-Cep63 antibody (purchased from Millipore), and a consistent result was obtained. Arrow and arrowheads indicate mature centrosome and extra Cep63 dots, respectively. Scale bar = 5 µm.

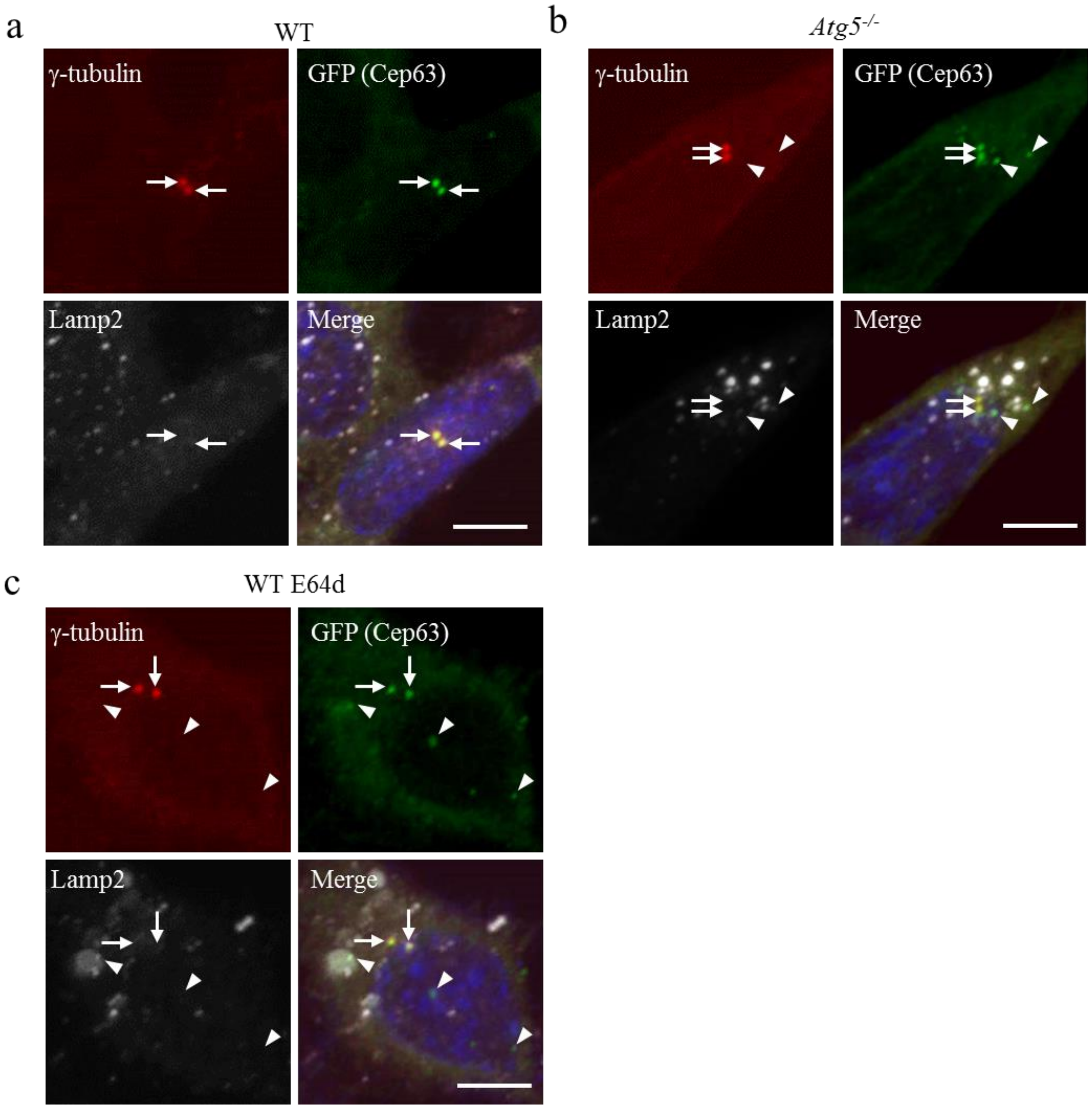

Supplementary Figure 6 Examination of extra Cep63 dots using GFP–Cep63.

The same experiments as shown in Figure 2 **(a-c)** were performed using exogenously expressed GFP–Cep63 instead of Cep63 immunostaining. Arrows and arrowheads indicate mature centrosomes and extra Cep63 dots, respectively. Scale bar = 5 μm.

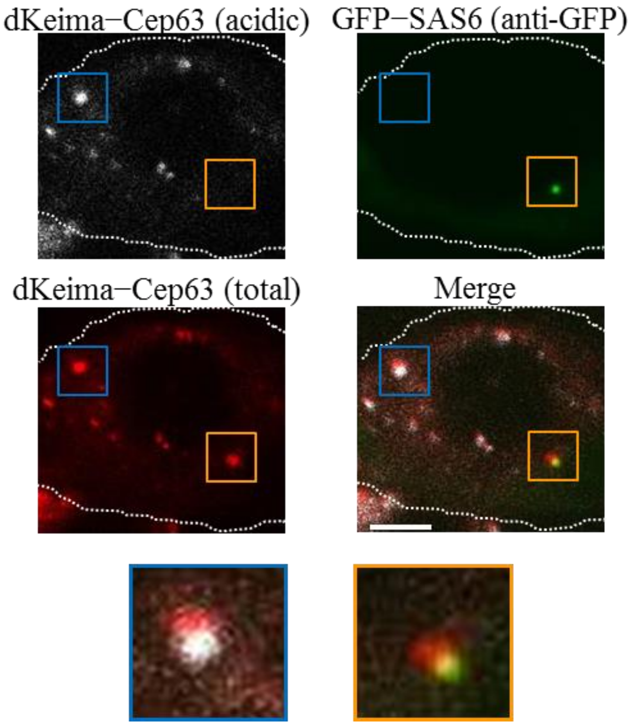

Supplementary Figure 7 Regulation of extra Cep63 dots by autophagy.

WT MEFs were transfected with dKeima–Cep63 and GFP–SAS6 plasmids. Representative images of acidic dKeima–Cep63 (white; upper left), total dKeima–Cep63 (red; lower left), GFP–SAS6 immunostained with an anti-GFP antibody (green; upper right), and a merged image (lower right) are shown. Centrosomes containing GFP–SAS6 did not show the acidic dKeima signal (blue squares), whereas extra Cep63 dots without the presence of SAS6 showed acidic signals (orange squares). Magnified images of the area within the blue and orange squares are shown below. White lines indicate the cell shape. Because there is the possibility that GFP fluorescence is quenched in acidic compartments, the presence of GFP–SAS6 was evaluated by immunostaining using an anti-GFP antibody. Scale bar = 5 µm.

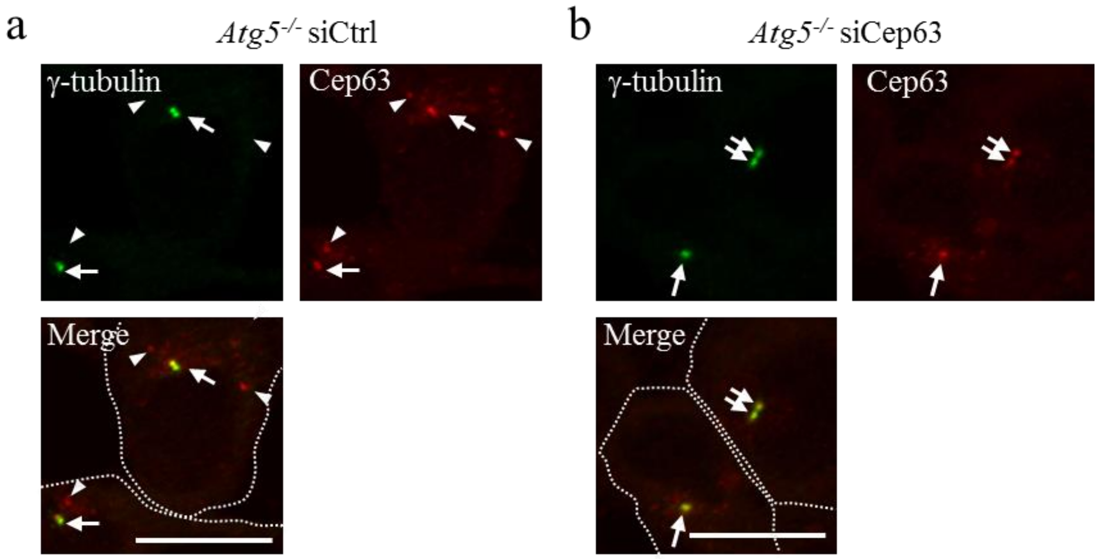

Supplementary Figure 8 Regulation of centrosome number by Cep63.

Atg5$^{-/-}$ MEFs were transfected with Cep63 siRNA (siCep63) and scramble siRNA (siCtrl) for 24 h. After 24 h, the cells were immunostained with anti-γ-tubulin and anti-Cep63 antibodies and were examined by fluorescence microscopy. Representative images of γ-tubulin (green; upper left), Cep63 (red; upper right), and the merged image (lower left) are shown. Arrows and arrowheads indicate mature centrosomes and extra Cep63 dots, respectively. White dotted lines indicate the cell shape. Scale bar = 10 μm.

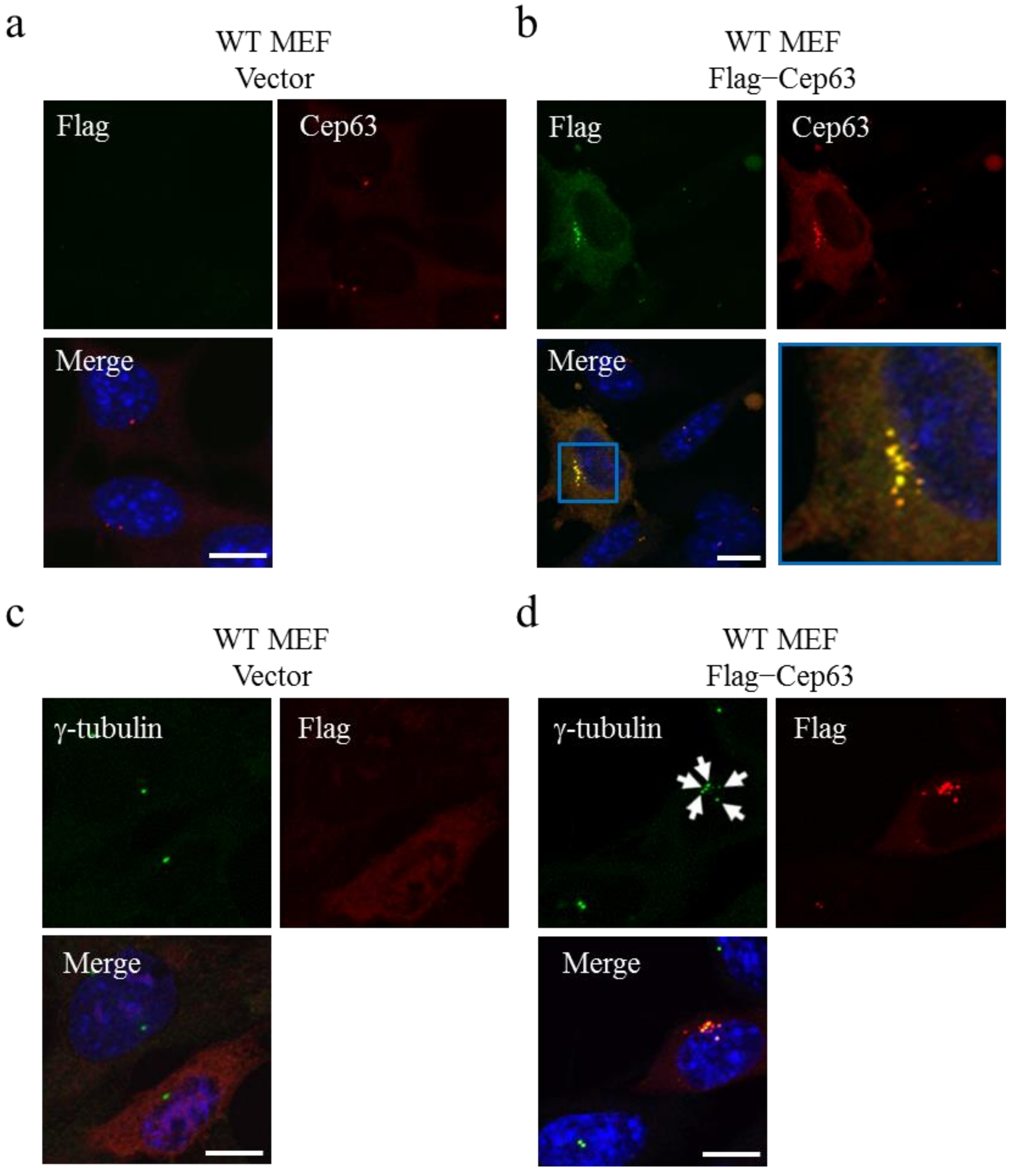

Supplementary Figure 9 Overexpression of Cep63 increases centrosome number.

WT MEFs were transfected with Flag–Cep63 and a control vector for 24 h. The cells were then immunostained with anti-Flag **(a–d)**, anti-Cep63 **(a, b)**, and anti-γ-tubulin **(c, d)** antibodies and were examined by fluorescence microscopy. Nuclei were stained with DAPI. In **(a, b)**, representative images of Flag (green; upper left), Cep63 (red; upper right), and a merged image (lower left) are shown. Scale bar = 10 μm. In **(b)** a magnified image of the area within the blue square is shown at the right. An increased number of Cep63 dots were observed in the Flag–Cep63-transfected wild-type MEFs. In **(c, d)**, representative images of γ-tubulin (green; upper left), Flag (red; upper right), and a merged image (lower left) are shown. Arrows indicate mature centrosomes. Scale bar = 10 μm. Representative Flag–Cep63-transfected MEFs, but not control MEFs, contained three or more centrosomes.

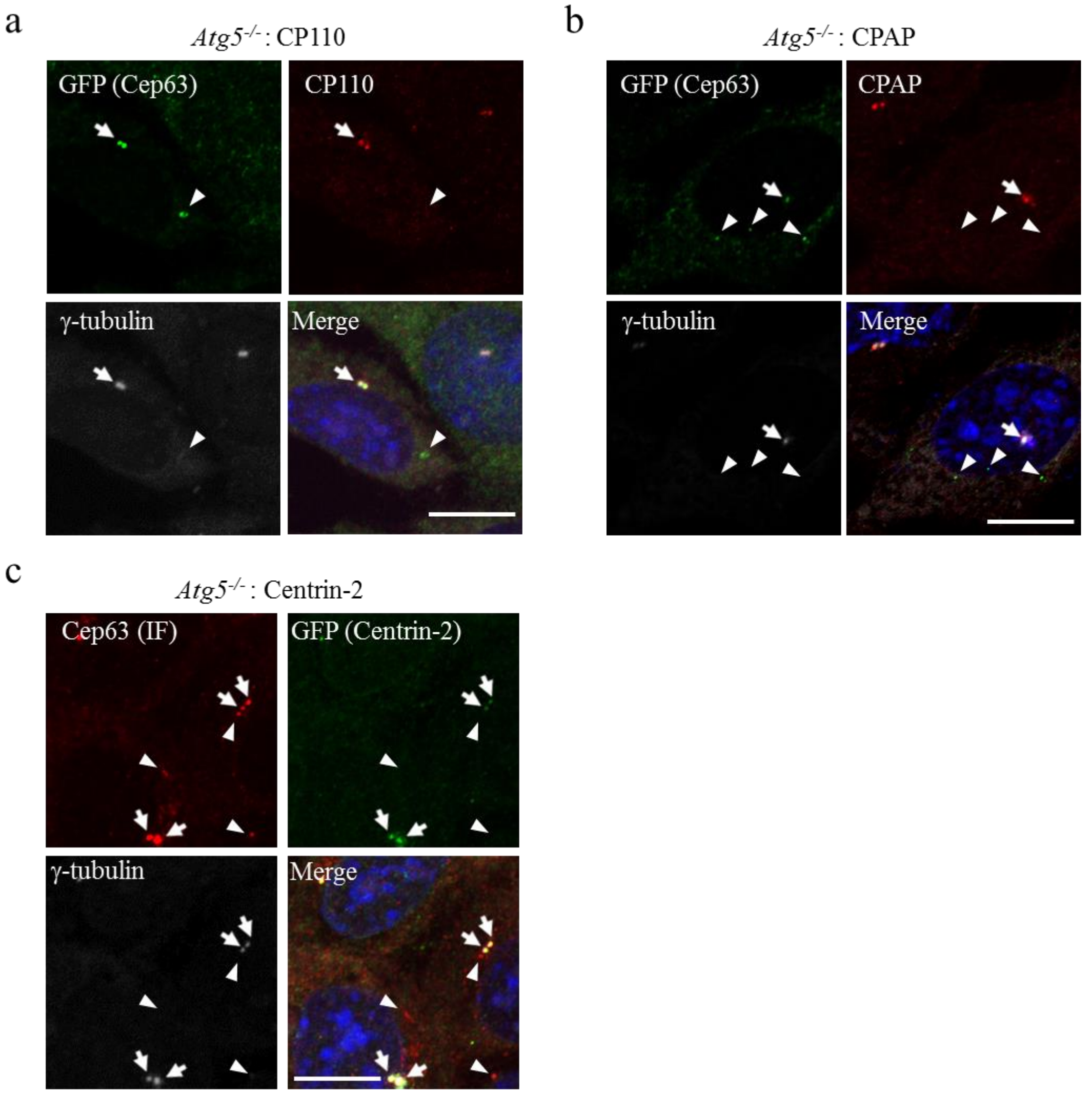

Supplementary Figure 10 No co-localization of CP110, CPAP, and Centrin-2 with extra Cep63 dots.

(**a**, **b**) *Atg5*$^{-/-}$ MEFs transfected with GFP–Cep63 were immunostained with anti-γ-tubulin, anti-GFP, and anti-CP110 **(a)** or anti-CPAP **(b)** antibodies and were examined by fluorescence microscopy. The representative images of Cep63 (green; upper left), CP110 or CPAP (red; upper right), γ-tubulin (white; lower left), and the merged image (lower right) are shown. **(c)** *Atg5*$^{-/-}$ MEFs transfected with GFP-centrin-2 were immunostained with anti-γ-tubulin, anti-GFP, and anti-Cep63 antibodies and were examined by fluorescence microscopy. Representative images of Cep63 (red; upper left), centrin-2 (green; upper right), γ-tubulin (white; lower left), and the merged image (lower right) are shown. Arrows and arrowheads indicate mature centrosomes and extra Cep63 dots, respectively. The nucleus is stained with DAPI. Scale bar = 10 µm.

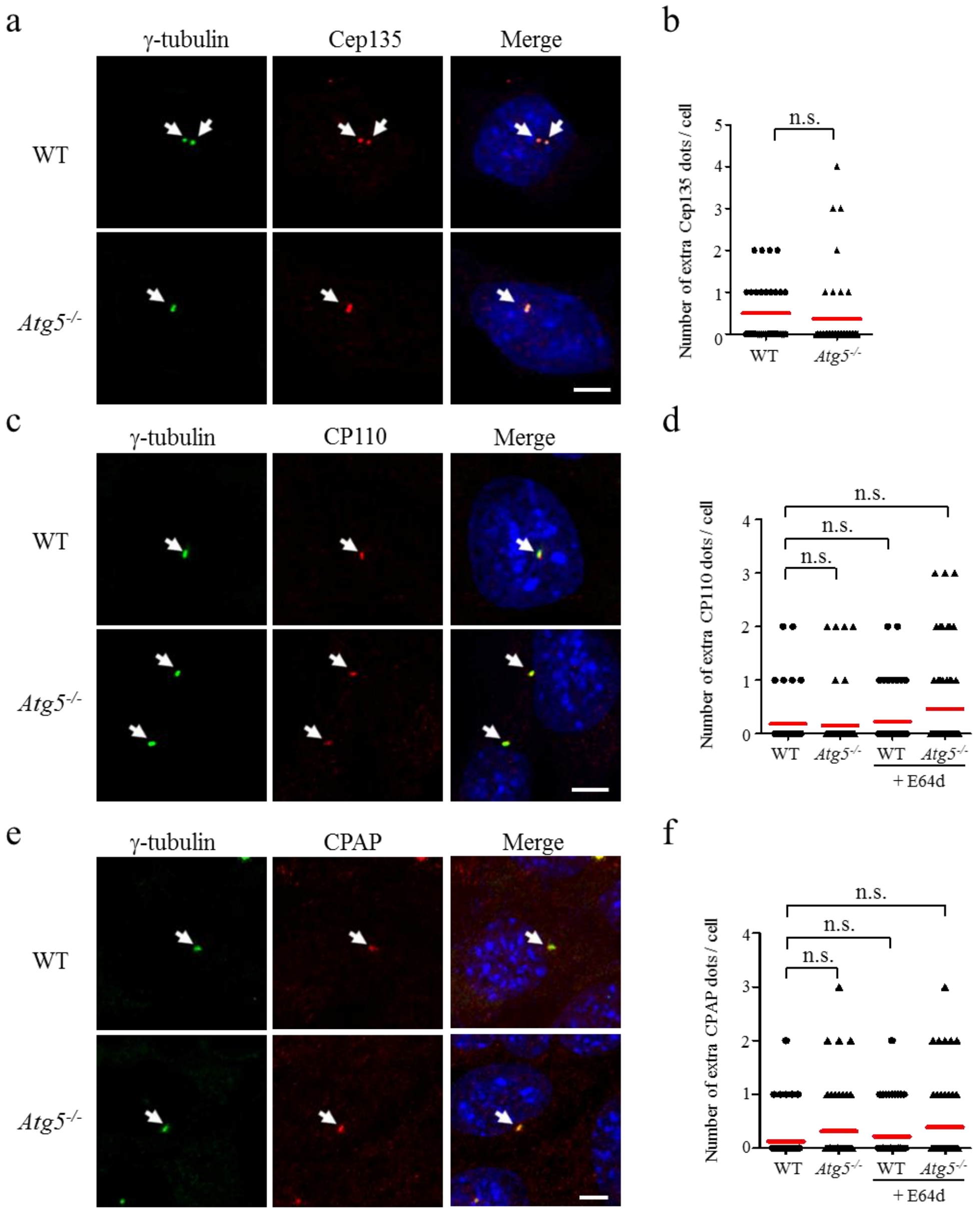

Supplementary Figure 11 No difference of extra dots in Cep135, CP110, and CPAP between wild-type MEFs and *Atg5*$^{-/-}$ MEFs.

Wild-type (WT) MEFs and *Atg5*$^{-/-}$ MEFs in the presence or absence of E64d were immunostained with anti-γ-tubulin and anti-Cep135 (**a**, **b**), anti-CP110 (**c**, **d**), and anti-CPAP (**e**, **f**) antibodies and were examined by fluorescence microscopy. Representative images of γ-tubulin (green; left) and Cep135, CP110, or CPAP (red; middle), and the merged image (right) are shown in (**a, c** and **e**). Arrows indicate mature centrosomes. The nucleus is stained with DAPI. Scale bar = 5 μm. (**b, d** and **f**) The number of extra Cep135 (**b**), extra CP110 (**d**), and extra CPAP (**f**) dots per cell was calculated (n > 30 cells). Red lines indicate mean values. "n.s." indicates no significant difference (ANOVA Turkey's *post hoc* test).

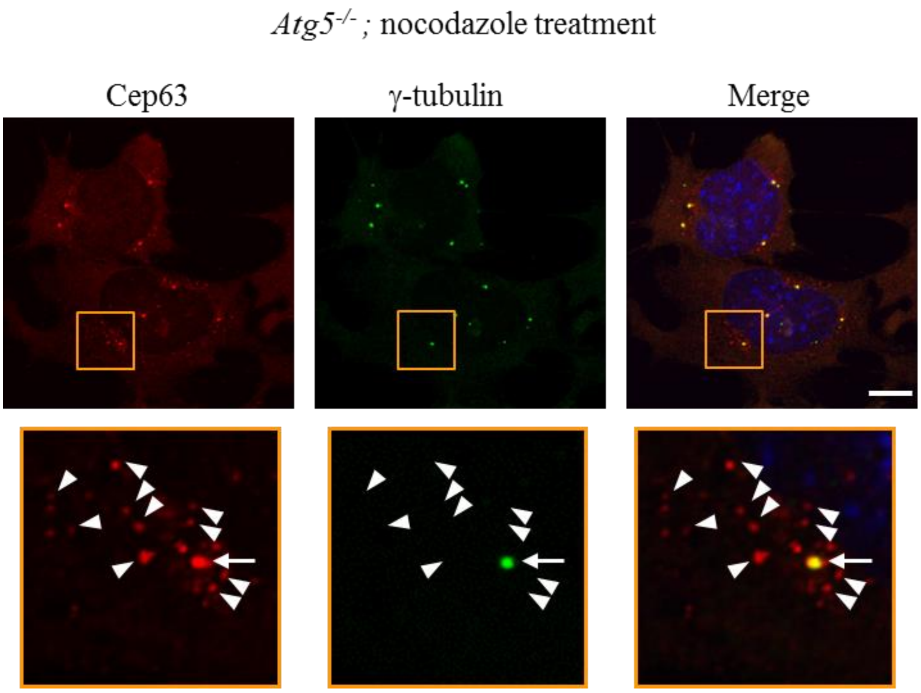

Supplementary Figure 12 Concentration of extra Cep63 dots surrounding the mother centriole by nocodazole treatment.

Atg5$^{-/-}$ MEFs were treated with nocodazole (100 nM) for 24 h. The cells were then immunostained with anti-Cep63 and anti-γ-tubulin antibodies and were examined by fluorescence microscopy. The representative images of Cep63 (red; left), γ-tubulin (green; center) and the merged image (right) are shown. Nuclei are stained with DAPI. Scale bar = 10 μm. Magnified images in the squares are shown below. Arrows and arrowheads indicate mature centrosomes and extra Cep63 dots, respectively.

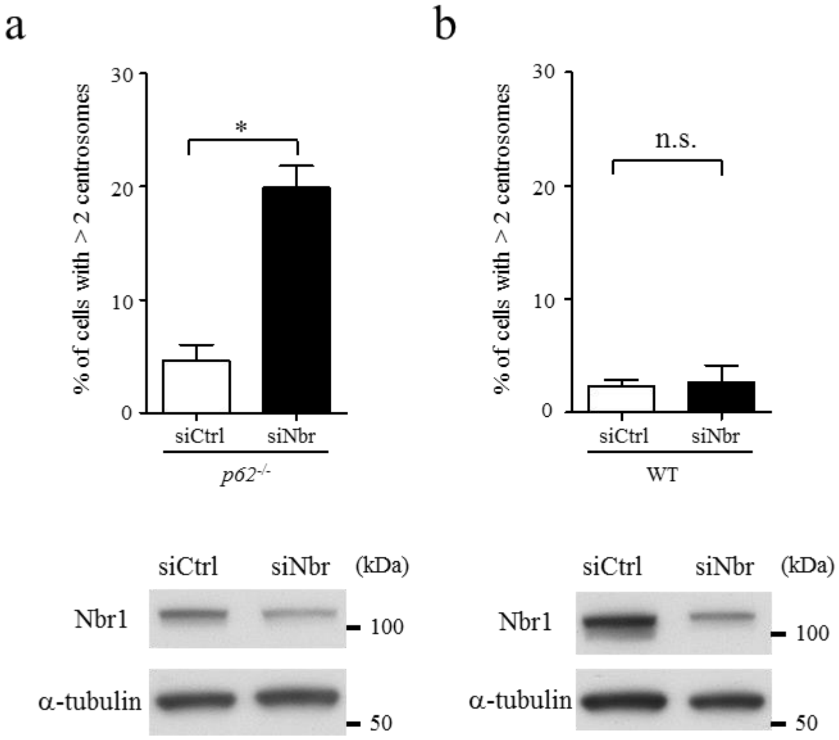

Supplementary Figure 13 Nbr1 compensates for the absence of p62.

p62$^{-/-}$ MEFs **(a)** and wild-type MEFs **(b)** transfected with Nbr1 siRNA (siNbr) or scramble siRNA (siCtrl) for 48 hr were immunostained with an anti-γ-tubulin antibody. The percentage of cells with three or more centrosomes was calculated. Data are shown as the mean + s.d. (n = 3). The asterisk indicates a statistically significant difference (p < 0.05). "n.s." indicates no significant difference (Student's *t*-test). An efficient reduction in Nbr1 level was confirmed by western blotting using an anti-Nbr1 antibody. Uncropped images are shown in Supplementary Fig. 14.

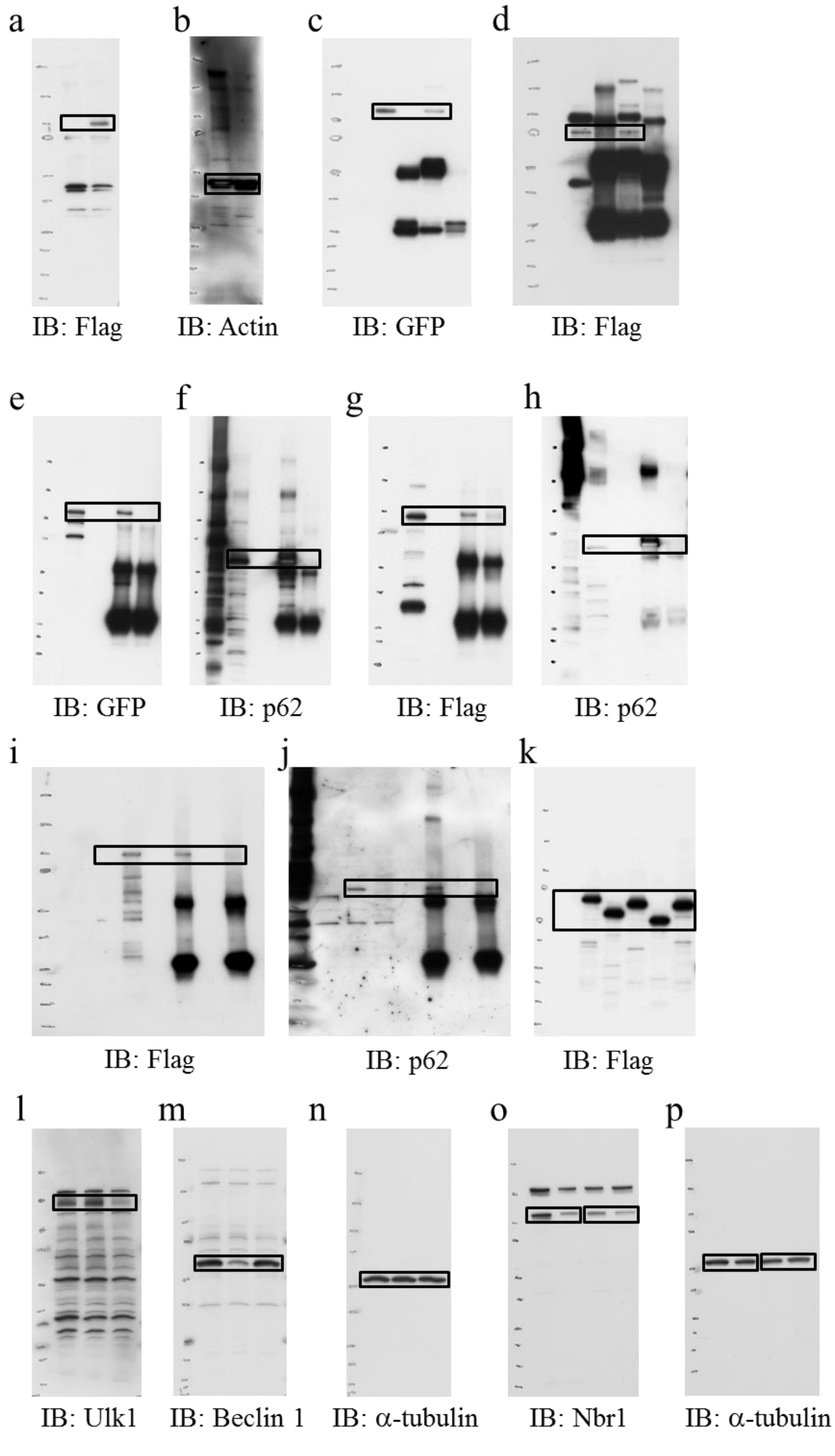

Supplementary Figure 14 Whole blot images
